# Poster Session II - A222 STUDIES BASED ON HEALTH ADMINISTRATIVE DATA REGARDING RARE OUTCOMES IN INFLAMMATORY BOWEL DISEASE SIGNIFICANTLY UNDERESTIMATE THE TRUE RISK - THE IMPORTANCE OF SPECIFICITY

**DOI:** 10.1093/jcag/gwaf042.222

**Published:** 2026-02-13

**Authors:** M Malham, E Benchimol, M Fox, D Wilson

**Affiliations:** Division of Gastroenterology, Hepatology and Nutrition, Department of Paediatrics, University of Toronto, Toronto, ON, Canada; Division of Gastroenterology, Hepatology and Nutrition, Department of Paediatrics, University of Toronto, Toronto, ON, Canada; Boston University, Boston, MA; The University of Edinburgh College of Medicine and Veterinary Medicine, Edinburgh, Scotland, United Kingdom

## Abstract

**Background:**

Health administrative data (HAD) has increased our knowledge of rare outcomes in inflammatory bowel disease (IBD), such as cancer and mortality. However, most neglect to address the vulnerability to systematic misclassification of IBD diagnosis (information bias)

**Aims:**

We aimed to assess the information bias imposed by misclassification of the IBD diagnosis in HAD studies by performing quantitative bias analysis (QBA).

**Methods:**

We identified pediatric-onset IBD (PIBD) HAD studies assessing cancer risk in which the PIBD case identification was based on published validation studies. We then performed probabilistic QBA to adjust for non-differential exposure misclassification using the sensitivity and specificity values from country or region-specific validation studies.

**Results:**

We included four studies presented in Table 1, along with reported and bias-adjusted outcome estimates. Of these studies, the Canadian and Danish studies, which had specificity values of 0.92 and 0.91, respectively, had the largest degree of misclassification bias, biasing the results towards the null. The bias-adjusted relative risks were two to three times higher than the reported results. The most considerable divergence was found in the Canadian study, with a reported relative risk of 2.0 (95%CI: 1.2-3.4) and a bias-adjusted relative risk of 5.8 (95%CI: 2.5-13.7). The corresponding RD was 1.0% (95%CI: 0.1-1.9) and the bias-adjusted RD was 3.8% (95%CI: 1.4-7.9).

**Conclusions:**

We exemplify the importance of supplementing HAD-based cohort studies with QBA. To do this, it is imperative that validation studies are available on case assertion algorithms within the specific registry for any disease group that a forthcoming study would be reporting on. Our results indicate that most HAD-based studies on rare long-term consequences of IBD significantly underestimate the true risk of the outcomes. Because the same underlying information bias exists in adult literature, our result can be extrapolated to HAD-based IBD studies in all age groups.

**Funding Agencies:**

None

**A222 Table 1:** 

Country	Cancer in IBD (yes/no)	Cancer in RI (yes/no)	Reported estimate (95%CI)	Range of adjusted sensitivity/specificity	Risk estimate adjusted for misclassification
Canada (Manitoba)	17/930	75/9,197	HR: 2.0 (1.2-3.4)RD: 1.0% (0.1-1.9)	(0.77-0.83)/(0.92-0.94)	RR: 5.80 (2.46-13.7)RD: 3.8% (1.4-7.9)
Denmark	158/6,222	701/53,099	HR: 2.2 (1.8 - 2.6)RD: 1.2% (0.8-1.6)	(0.81-0.89)/(0.91-0.94)	RR: 3.95 (2.69-6.78)RD: 3.7% (2.4-7.1)
Israel	14/3,944	14/12,991	RR: 3.3 (1.6 - 6.9)RD: 0.25% (0.05-0.44)	(0.87-0.92)/(0.99-1.0)	RR: 3.67 (1.62-8.77)RD: 0.26% (0.05-0.48)
Sweden	497/8,908	2,256/90,614	HR: 2.2 (2.0 - 2.5)RD: 2.90% (2.40-3.32)	(0.83-0.92)/(1.0-1.0)	RR: 2.21 (2.00-2.45)RD: 2.9% (2.4-3.4)

HR, Hazard ratio. RR, relative risk. RD, risk difference.

